# Personalized redox medicine in inflammatory bowel diseases: an emerging role for HIF-1α and NRF2 as therapeutic targets

**DOI:** 10.1016/j.redox.2023.102603

**Published:** 2023-01-06

**Authors:** Arno R. Bourgonje, Damian Kloska, Anna Grochot-Przęczek, Martin Feelisch, Antonio Cuadrado, Harry van Goor

**Affiliations:** a Department of Gastroenterology and Hepatology, University of Groningen, University Medical Center Groningen, Groningen, the Netherlands; b Malopolska Centre of Biotechnology, Jagiellonian University, Kraków, Poland; c Department of Medical Biotechnology, Faculty of Biochemistry, Biophysics and Biotechnology, Jagiellonian University, Kraków, Poland; d Clinical and Experimental Sciences, Faculty of Medicine, University of Southampton, Southampton, United Kingdom; e Instituto de Investigaciones Biomédicas “Alberto Sols” UAM-CSIC. Centro de Investigación Biomédica en Red Sobre Enfermedades Neurodegenerativas (CIBERNED), ISCIII, Madrid, Spain; f Department of Pathology and Medical Biology, University of Groningen, University Medical Center Groningen, Groningen, the Netherlands

**Keywords:** Inflammatory bowel disease, Redox medicine, Oxidative stress, HIF-1α, NRF2

## Abstract

Inflammatory bowel diseases (IBD), encompassing Crohn's disease (CD) and ulcerative colitis (UC), are intimately associated with inflammation and overproduction of reactive oxygen species (ROS). Temporal and inter-individual variabilities in disease activity and response to therapy pose significant challenges to diagnosis and patient care. Discovery and validation of truly integrative biomarkers would benefit from embracing redox metabolomics approaches with prioritization of central regulatory hubs. We here make a case for applying a *personalized redox medicine* approach that aims to selectively inhibit pathological overproduction and/or altered expression of specific enzymatic sources of ROS without compromising physiological function. To this end, improved ‘clinical-omics integration’ may help to better understand which particular redox signaling pathways are disrupted in what patient. Pharmacological interventions capable of activating endogenous antioxidant defense systems may represent viable therapeutic options to restore local/systemic redox status, with HIF-1α and NRF2 holding particular promise in this context. Achieving the implementation of clinically meaningful mechanism-based biomarkers requires development of easy-to-use, robust and cost-effective tools for secure diagnosis and monitoring of treatment efficacy. Ultimately, matching redox-directed pharmacological interventions to individual patient phenotypes using predictive biomarkers may offer new opportunities to break the therapeutic ceiling in IBD.

## Abbreviations

ARE: antioxidant response element
5-ASA: 5-aminosalicylic acid
CD: Crohn's disease
CRP: C-reactive protein
DMF: dimethyl fumarate
DMOG: dimethyloxallylglycine
DNBS: 2,4-dinitrobenzene sulfonic acid
DSS: dextran sulfate sodium
FCal: fecal calprotectin
FIH: factor inhibiting HIF
GI: gastrointestinal
HIF-1α: hypoxia-inducible factor 1α
HRE: hypoxia response element
IBD: inflammatory bowel diseases
KEAP1: Kelch-like ECH-associated protein 1
LMW: low-molecular-weight
NADPH: nicotinamide adenine dinucleotide phosphate
NRF2: nuclear factor erythroid 2-related factor 2
p300/CBP: acetyltransferases p300 and CREB binding protein
PHD: prolyl hydroxylase
RNS: reactive nitrogen species
ROS: reactive oxygen species
RSI: reactive species interactome
RSS: reactive sulfur species
SFN: sulforaphane
TNBS: 2,4,6-trinitrobenzene sulfonic acid
UC: ulcerative colitis
VHL: von Hippel-Lindau tumor suppressor protein

## 1 Inflammatory bowel diseases and personalized redox medicine

Inflammatory bowel diseases (IBD), encompassing Crohn's disease (CD) and ulcerative colitis (UC), are complex, heterogeneous, immune-mediated diseases of the gastrointestinal (GI) tract [1]. Multiple pathophysiological processes play a role in their onset and progression, driven by a complex interplay between genetic susceptibility, gut microbiota, host immunity, and the aggregate of lifestyle, diet, and environment-related factors collectively referred to as the ‘exposome’ [2,3]. The ‘exposome’ comprises the biological responses to physical factors and synthetic chemicals in our environment, as well as to dietary constituents and psychosocial stressors from conception to death, all of which are considered to contribute to the development of chronic metabolic inflammation (*metaflammation*) [4,5]. Although our knowledge about exposome–driven pathophysiological processes is advancing rapidly, considerable knowledge gaps remain regarding disease heterogeneity, e.g. why patients experience alternating episodes of quiescent periods with mild or even absent symptoms and overt disease. Furthermore, the high degree of complexity and heterogeneity also contributes to the difficulty to detect the disease early, monitor disease activity as well as predict therapeutic responses and prognosis using existing clinical knowledge/tools. As such, there is an urgent need for novel *biomarkers* in IBD. Biomarkers are commonly defined as objectively measured indicators of (ab)normal biological processes/systems or pharmacological responses to therapeutic interventions [6,7].

IBD is clinically characterized by symptoms like fatigue, abdominal pain, diarrhea, and weight loss, as well as extra-intestinal manifestations such as arthralgia, uveitis, and skin abnormalities, with considerable inter-individual variability. Currently, IBD diagnosis is based on a combination of clinical, biochemical and histologic criteria [8], and an invasive endoscopy procedure is warranted to establish a definitive diagnosis. While CD is characterized by discontinuous, patchy, and ulcerative inflammation that may occur throughout the entire GI tract, UC is marked by rather superficial inflammation limited to the colon [9,10]. Currently, there is no cure for IBD, and the majority of patients need lifelong immunosuppressive treatment to control inflammation and minimize the risk of requiring surgical intervention.

Monitoring disease activity is an important component of clinical care for patients with IBD since long-lasting subclinical disease activity increases the risk of developing disease complications, hospitalization, and surgical resection of (part of) the bowel [11]. Importantly, it also negatively influences patients’ quality of life and social interactions [12]. Currently, biomarkers such as *C*-reactive protein (CRP) and fecal calprotectin (fCal) are used to detect and monitor disease activity. However, these biomarkers demonstrate inconsistent associations with mucosal inflammation, have limited specificity, and limited predictive capacity for long-term disease outcome [[13], [14], [15]]. This underscores the need for disease biomarkers capable of predicting the future occurrence of exacerbations.

IBD is characterized by a disturbed gut mucosal homeostasis, with distinct immunological alterations and gut microbial disturbances [16]. Active intestinal inflammation is accompanied by an overproduction of reactive oxygen species (ROS), culminating in oxidative stress as a key pathophysiological driver for IBD development [17]. Oxidative stress is defined as an imbalance between oxidants and antioxidants in favor of the oxidants, disrupting redox signaling and control and leading to molecular damage [18]. However, physiologically ROS also serve important signaling functions in immune cells [19]. Excessive ROS production by an inflamed intestinal mucosa can lead to oxidative damage, perpetuate the inflammatory process and give rise to fibrosis, tissue destruction, or both [20]. In the context of IBD, oxidative stress is not only limited to the intestinal mucosa, but extends to the whole-body redox system, and is thus mirrored within the systemic circulation [21], offering an opportunity for minimally invasive quantification of circulating contributors to the systemic redox status. Besides ROS, also other types of short-lived compounds including reactive nitrogen (RNS) and reactive sulfur species (RSS) contribute to the signaling processes and merit further consideration. This has recently been conceptualized in the so-called ‘Reactive Species Interactome’ (RSI) framework [22]. The RSI provides a useful structure within which considerations such as *personalized redox medicine* could be organized across different levels of biological organization.

A biomarker-driven *systems biology* approach has increasingly been advocated to improve our understanding of IBD pathogenesis and inter-individual biological variability [23]. Systems biology purports to provide holistic mathematical modeling of complex biological systems [24]. With the advent of multi-omics technologies, e.g. transcriptomics, proteomics, and metabolomics, unprecedented insights derived from multiple layers of biological organization have become available; in combination with computational advances in e.g. machine learning and artificial intelligence this may facilitate the integration of ‘big data’ and eventually enable the establishment of molecular constructs that are specific and clinically useful for IBD [25]. To achieve this goal, it will be critical to provide highly specific information that integrates global biological analysis of each individual patient (‘precision medicine’) and clinical decision-making. The interrogation of such immensely complex molecular data constructs has to be accompanied by careful curation of clinical information about patient characteristics, clearly defined endpoints, and independent verification of biomarker signatures. Given the emergence of a number of redox-targeted therapeutics in the IBD field (e.g. hypoxia-inducible factor 1α (HIF-1α)-agonists/stabilizers or nuclear factor erythroid 2-related factor 2 (NRF2)/Kelch-like ECH-associated protein 1 (KEAP1) modulators), these novel treatments should be assessed in terms of effectiveness, safety, and tolerability at the individual patient level.

As ‘master regulator’ of oxygen handling the hypoxia-inducible factor (HIF)-1α pathway plays a critical role in maintaining intestinal epithelial homeostasis [26]. In IBD, the ability of the organism to cope with physiological levels of hypoxia is overwhelmed by the much greater degree of pathophysiological hypoxia due to intestinal inflammation [27]. HIF controls the resultant level of oxidative stress and tissue hypoxia by activating numerous cellular target genes to counteract this situation. Accumulating evidence supports a protective role for HIF-1α in response to pathophysiological hypoxia, and, additionally, in attenuating intestinal inflammation and enhancing intestinal barrier integrity [28]. Currently, pharmacological agents targeting the HIF-1α pathway are being evaluated in early-phase clinical trials. The NRF2/KEAP1 pathway, sometimes considered to be the ‘master regulator’ of cellular antioxidant defense, is another emerging target of interest in the context of IBD because of its role in combating oxidative stress in disease progression. Under physiological conditions, KEAP1 negatively regulates NRF2 activity. Oxidative modifications to the cysteine residues in KEAP1 lead to conformational protein changes that release NRF2 from KEAP1-mediated suppression. This, in turn, leads to the translocation of NRF2 to the cell nucleus and activation of downstream target genes to counteract oxidative (and electrophilic) cellular stress. Previous studies have demonstrated the protective effects of activating the NRF2/KEAP1 pathway through decreasing intestinal inflammation, upregulating antioxidant defense, and protecting the intestinal epithelial barrier, providing a clear rationale for therapeutic modulation [29]. Thus, NRF2 activation could be a viable therapeutic strategy to mitigate intestinal inflammation, with several compounds currently being evaluated [30]. Of note, both HIF-1 and NRF2 are targets as well as effectors of redox signaling/regulation [27,29]; moreover, both transcription factors are continuously produced just to be immediately degraded via the ubiquitin-proteasome pathway, attesting to their significance for resilience.

In this review, trends and perspectives on the development of *personalized redox medicine* for IBD will be outlined, aimed at the selective inhibition of pathological overproduction and/or expression of specific enzymatic sources of reactive species without compromising their physiological roles. First, we will review the role of disrupted redox signaling in IBD, followed by a critical appraisal of the utility of redox biomarkers and recent advances in this field, with a focus on integrative biomarkers. Subsequently, the emerging therapeutic targets HIF-1α and NRF2/KEAP1 will be discussed as potential therapeutic options with regard to their ability to restore impaired redox status in IBD. To this end, a more nuanced understanding of disrupted redox signaling in this disease setting will be required, alongside translational efforts to achieve true ‘clinical -omics integration’.

## 2 Moving from oxidative stress to redox medicine in IBD

### 2.1 Oxidative stress and disrupted redox signaling in the pathophysiology of IBD

In addition to oxidative stress due to chronic intestinal inflammation, IBD is also associated with a disruption of redox signaling [9]. Redox imbalance is considered an important pathophysiological effector mechanism that may inflict cellular/molecular damage and injury to the inflamed intestinal mucosa [31]. In IBD, the inflamed gut mucosa is characterized by infiltration of a vast number of inflammatory cells, accompanied by the production of (pro)inflammatory mediators and the failure of existing anti-inflammatory defense mechanisms (e.g. regulatory cells, anti-inflammatory cytokines, antimicrobial peptides, antioxidants) to preserve tissue homeostasis [32]. The first evidence for an existing redox imbalance was derived from *ex vivo* studies of affected intestinal tissue [17,[33], [34], [35]]. Active inflammatory cells that infiltrate the intestinal mucosa lead to the production and release of large fluxes of ROS, crucial for host defense and pathogen killing. In addition, intestinal inflammation impairs mitochondrial function, and in spite of enhanced angiogenesis and increased vascular density results in a situation where oxygen cannot be utilized in the tissues (metabolic dysoxia), leading to further ROS production through activation of the hypoxia-inducible factor (HIF) transcription factor family [36]. Oxidative stress and perturbation of physiological redox signaling have been linked to the onset of IBD and related disease manifestations [17,[37], [38], [39]]. ROS production by immune cells has been suggested to already occur before the actual infiltration of the intestinal mucosa by these cells, substantiating the relevance of these processes to pathophysiology [40]. Furthermore, the continuous exposure of the intestinal mucosa to oxidative damage compromises gastrointestinal functioning, which may include, but is not limited to, intestinal malabsorption, dysmotility, inadequate electrolyte handling, and disrupted integrity of the GI barrier through epithelial cell injury [[41], [42], [43]].

### 2.2 The concept of ‘oxidative stress’ needs refinement

Historically, the concept of oxidative stress has been defined as a disturbance in the pro-oxidant/antioxidant system in favor of the former [44]. Local disruption of this balance may occur due to inflammatory processes, which are accompanied by immune cell activation (particularly first-line innate defenders such as granulocytes and macrophages), and by the NOX2-mediated ‘oxidative burst’ leading to targeted production of ROS. If persisting, such local events (ranging from molecular responses to oxidative damage of the surrounding tissues) can surpass the available ROS-scavenging capacity with a consequent disequilibrium in the rates of ROS formation (e.g. by increased activity of NADPH oxidases or as byproducts of aerobic metabolism) and neutralization (e.g. through antioxidant enzymes such as superoxide dismutase and/or catalase). These events may occur in tissues or be even localized to specific cellular compartments only. In contrast, numerous other redox signaling events are required to operate normally to fulfill essential cellular physiologic processes, e.g. cell growth, differentiation, proliferation, and apoptosis [45], yet this may become challenging under conditions of chronic ROS overproduction. Furthermore, ROS are physiologically relevant for immunological and mitochondrial functioning [46] and mediate autophagy in response to cellular stress [47]. Importantly, when considering pathological dysregulation of ROS, this does not necessarily relate to large quantities being produced but may also occur due to a pathological shift in location (e.g. ROS formation at sites where physiologically little ROS formation is expected) or regulation (e.g. a specific redox-sensitive regulatory enzyme being disturbed or ROS formation interfering with redox-sensitive metabolic pathways) [48]. Although the concept of oxidative stress has prevailed for years and is still commonly referred to today, an updated definition has recently been suggested [49]. This includes a better distinction between the physiological and pathological formation of ROS with differentiation between “oxidative eustress” and “oxidative distress”. The idea that ROS are generally ‘bad’ and antioxidants are ‘good’ is severely outdated and does not appreciate the wealth of knowledge gained about redox biology over the past few decades. Clinical trials testing the effects of antioxidants have not provided support for this overly simplistic view, and in most settings (including IBD) compounds tested failed to demonstrate beneficial effects [50].

### 2.3 Antioxidants as an adjunct therapy in IBD: lessons learnt and future goals

Antioxidant therapy would seem to be a logical first choice for the treatment of IBD given the significant role of oxidative stress in its pathophysiology [51]. In fact, aminosalicylate (5-ASA) drugs, commonly prescribed for patients with UC, act at least partially by attenuating oxidative stress since they can scavenge reactive species [52]. For instance, they inhibit peroxynitrite (ONOO^−^)-mediated reactive species formation [53] and abrogate the aberrant formation of reactive nitrogen species (RNS) in experimental animal models such as dextran sulfate sodium (DSS)-induced colitis [54]. A limited number of randomized controlled trials are available that investigated the therapeutic potential of antioxidants in IBD. In patients with UC, studies have tested the effects of *N*-acetylcysteine and curcumin supplementation as an add-on to aminosalicylate therapy and observed improvement in disease activity [55,56]. Another study investigated the potential effect of combined supplementation of fish oil and vitamins A, –C, -E, and selenium and observed decreased production of pro-inflammatory cytokines [57]. Although beneficial, albeit moderate effects have been observed, most trials used relatively short follow-up periods, low-potency antioxidants, relatively low doses of antioxidants, and patient groups with either quiescent or mild disease activity. Considering these limitations, there is a clear need for future research in this field, especially since most studies investigating the therapeutic effects of antioxidants in IBD were not randomized or placebo-controlled, and often conducted in relatively small, heterogeneous patient cohorts. Other reasons may contribute to the absence of therapeutic failure of antioxidant therapies. For instance, reactive species fulfill essential physiological functions, implying that antioxidant interventions may potentially impair those functions. Theoretically, transport signaling processes dependent on disulfide bonds in membrane proteins, disequilibria in circulating thiol/disulfide balances, and single-electron oxidation of thiols may occur giving rise to the formation of thiyl radicals [[58], [59], [60]]. Either one of these processes may be disrupted by excessive scavenging of reactive species following the administration of an antioxidant (especially in patients who do not suffer from an overproduction of such species). It would therefore seem to be critical to carefully interrogate an individual's redox phenotype to avoid interference with physiological redox signaling processes. Accurately defining the patient-specific level of “redox stresses'' could offer therapeutic decisions to become more personalized by increasing the chances of achieving an overall whole-body redox balance [61]. Following this rationale, one would aim to selectively inhibit only disease-relevant overproduction of reactive species while leaving the physiological reactive species signaling processes intact. How to achieve this is not entirely clear at present. Finally, identifying therapeutic targets remains challenging since there is still an insufficient understanding of the complex human redox signaling network [62]. Nevertheless, in this way, one might be able to improve the effectiveness, safety, and tolerability of therapeutic approaches and minimize the risk of imposing additional perturbations to the redox system. To this end, it has recently been suggested that a systems medicine or network pharmacology-based approach could help to achieve these goals [48].

### 2.4 Towards the use of ‘redox medicine’ in IBD

The concept of ‘redox medicine’ refers to all interventions in the healthcare process that favourably modulate disrupted redox signaling processes to improve redox balance. From a systems biology perspective, this emerging concept fits the paradigm of ‘personalized medicine’, which purports to provide tailored therapeutic and/or preventive strategies to combat disease (or, where this is already established, halts further disease progression). The RSI with all its different constituents may provide the conceptual framework to develop such redox-based approaches (see also section: ‘Clinical implementation of redox medicine in IBD: challenges and perspectives’). In-depth characterization of an individual's pheno/chemotype in conjunction with lifestyle-, dietary-, and environmental factors will be required to allow stratification of redox-derived biomarkers. Such individual redox biomarker signatures [63] could then function as decision support tools for therapy selection while considering pertinent risk factors. Concomitant profiling of whole-body RSI/redox status in the clinical context will be instrumental for the development of risk stratification tools and therapeutic modulation of targeted redox signaling nodes, including HIF1a and NRF2/KEAP1. We consider such an approach to be crucial for complex and heterogeneous diseases like IBD by offering guidance to therapeutic intervention along various stages of the disease process, including the prevention of complications.

## 3 Redox biomarkers in IBD: from damage markers to whole-body redox status

Oxidative stress is considered a critical effector mechanism in the pathogenesis of IBD, inflicting cellular and molecular damage to the intestinal mucosa and, therefore, closely associated with onset and progression of IBD. Most evidence for oxidative stress in IBD has been established by measurement of tissue- or blood-based biomarkers that represent either enhanced production of ROS or decreased availability of antioxidant compounds/enzyme activity, and these have been extensively reviewed previously [17,64]. As for the first category, studies have shown elevated levels of lipid-, protein-, or DNA-damage biomarkers, as well as elevated levels of oxidized biomarkers in which nitrogen- or sulfur compounds are represented. Regarding the antioxidant capacity, reduced levels of antioxidant enzymes, vitamins, or thiol/disulfide ratios of glutathione and other low-molecular-weight (LMW)-thiols have been reported. Although these studies indicated a prominent role of oxidative stress in IBD, the role of individual biomarkers within the redox signaling network and their relative contributions to stress has not been elucidated. These ‘damage’ biomarkers are often considered downstream end products originating from local inflammatory processes but many of these species are rather reactive themselves and capable of perturbing redox signaling at sites distant to the inflamed tissue, possibly explaining the systemic nature of the disease process. Our current lack of understanding of the many chemical interactions occurring *in vivo* across different levels of biological organization hampers the interpretation of existing redox measurements [62] and the development of specific ‘redox medicine’ approaches.

**Fig. 1:**
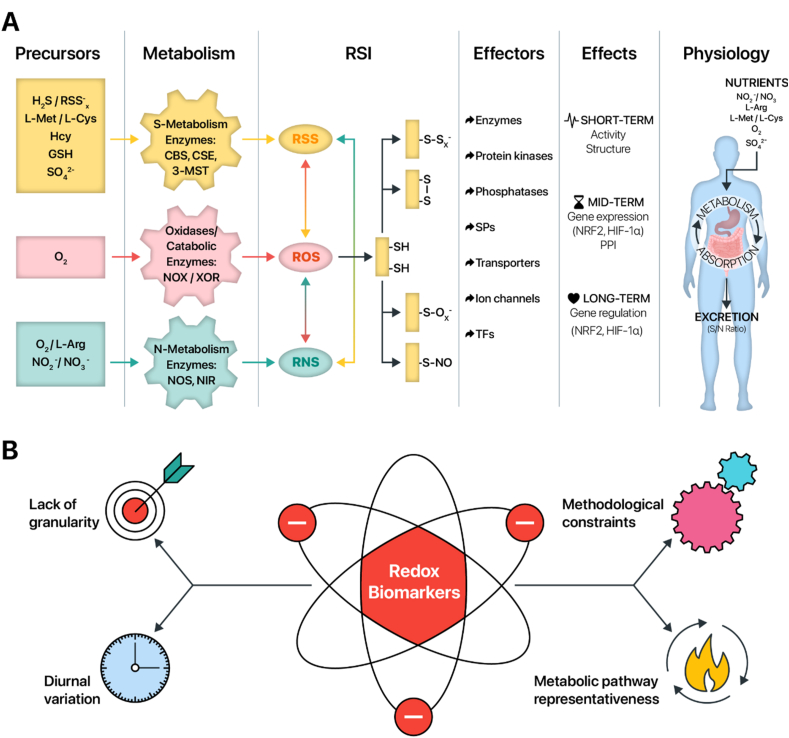
**A conceptual framework and key considerations to foster the development of redox biomarkers for whole-body redox status in IBD**. (**A**) Schematic overview of the key elements of the Reactive Species Interactome (RSI) which aims to describe chemical interactions among the different types of reactive species (ROS, RNS, and RSS) as well as their interactions with downstream biological targets, e.g. cysteine-based *redox switches*. Precursors of the RSI are mainly derived from the diet, consisting of organic (e.g. l-methionine, l-arginine) and inorganic (e.g. H_2_S, O_2_) compounds and cofactors (e.g. pyridoxal-5′-phosphate), which fuel intermediary metabolism and lead to the controlled formation of reactive species. The main biological targets of the RSI are cysteine-based redox switches, or thiols, acting as multimodal redox relays and modulating the activity of signaling molecules, leading to short-term (e.g. protein structure), mid-term (e.g. gene expression) and longer-term adaptations (e.g. gene regulation). Circulating levels of free thiols and stable end products of the RSI also serve as a communication conduit connecting the intestinal supply of RSI precursors with downstream intracellular thiol targets. (**B**) Key aspects considering the future of redox biomarkers in IBD, including a lack of granularity of biomarkers, methodological constraints, diurnal variation and their representativeness of redox-related metabolic pathways. Abbreviations: 3-MST, 3-mercaptopyruvate sulfurtransferase; CBS, cystathionine β-synthase; CSE, cystathionine γ-lyase; L-Arg, l-arginine; L-Cys, l-cysteine; L-Met, l-methionine; NIR, nitrite reductase; NOS, NO synthase; NOX, NADPH oxidases; RNS, reactive nitrogen species; ROS, reactive oxygen species; RSI, Reactive Species Interactome; RSS, reactive sulfur species; SPs, structural proteins; TFs, transcription factors; XOR, xanthine oxidoreductase.

A few years ago, efforts to conceptualize the intricate network of redox biology were integrated into a biological framework now known as the ‘Reactive Species Interactome’ (RSI) (Fig. 1). The RSI concept was established to describe the chemical interactions among different types of reactive species, i.e. ROS, RNS, and reactive sulfur species (RSS), as well as their downstream effects on biological targets including cysteine-based *redox switches* and metal centers [22]. Aside from the chemical interrelationships among these reactive species, the RSI also covers the metabolic pathways that are relevant to their endogenous generation through cellular intermediary metabolism, the transducing elements of redox regulation in the form of cysteine-based redox switches that influence intracellular redox targets, the adaptability to changes in metabolic demand and the capacity to sense and respond to changes in the extracellular environment. Oxidative modification of cysteine-based redox switches may give rise to a variety of short-, medium or long-term biological adaptations, which often involve post-translational modifications and are intimately linked to the global extracellular redox state [62] (Fig. 1A). At the level of the intestinal microenvironment, the RSI is fueled by the absorption and transport of nutritional (or synthetic) precursors of reactive species, including inorganic (e.g. oxygen, H_2_S) and organic (e.g. amino acids) compounds and cofactors (e.g. vitamins B_6_ and B_12_), which may also be derived from the gut microbiota. As such, the RSI may be considered a transducing entity that connects the organism's intestinal supply of RSI precursors with the full arsenal of intracellular thiol targets.

Aside from the metabolic representativeness of particular biomarkers of oxidative stress, there are also other challenges and unmet needs when attempting to identify clinically useful biomarkers (Fig. 1B). An important limitation pertains to the different technologies and methodological approaches adopted to quantify redox biomarker concentrations in biofluids. Often, the analytic determination is laborious and expensive, requiring sophisticated facilities to establish such measurements. One obvious reason for this is that direct quantification of reactive species is inherently difficult because of their very short biological half-lives since they are highly reactive and thus unstable [65]. Furthermore, their detection is cumbersome due to the multitude of chemical interactions in which they engage, the relatively low concentrations in biological fluids, and the lack of commercially available advanced detection kits. In spite of the use of rather sophisticated detection techniques, available methods often lack sensitivity and specificity to accurately determine physiological levels, thereby limiting data granularity. For example, only very few methodologies can discriminate between the different types of reactive species and their related (*S*-, *N*-, and O-derived) stable end products [66,67]. Taken together, this raises the question which molecules should be prioritized over others for redox phenotyping. Recently, recommendations for such integrated redox biomarkers have been proposed (see: *Clinical implementation of redox medicine in IBD: challenges and perspectives*).

Another aspect deserving consideration relates to the dynamic nature and diurnal variation of redox-related biomarkers, requiring longitudinal analysis of metabolic fluxes through specific redox-regulated pathways [68]. Longitudinal sampling across multiple time points in each individual allows capturing the temporal pattern of biomarker levels and may provide a more accurate characterization of the trajectories of underlying disease processes in IBD. In contrast, cross-sectional analysis and predictions from baseline fail to inform on the dynamics of regulation and cannot adequately reflect the complexity of the multi-layered redox regulatory network. A systematic longitudinal approach to biomarker analysis that captures their dynamics of regulation may aid in specifying the effects of physiological perturbations of the redox system. This way, differences due to circadian rhythms, pre- and postprandial levels, and the impact of exercise or varying levels of oxygen supply and consumption can be delineated. Likewise, the influence of chronic environmental stressors (e.g. living at high altitude with decreased inspired oxygen pressure as a result) could be taken into account. A recent study in a relatively small number of healthy individuals demonstrated that the combination of metabolic (moderate-intensity exercise) and environmental (low oxygen pressure) stressors resulted in distinct patterns of plasma concentrations of redox biomarkers irrespective of the fact that stressors were identical among participants [61]. Remarkably, this acute-on-chronic stress (exercise on top of low environmental oxygen pressure) resulted in dynamic alterations of concentrations upon onset and cessation, suggesting that redox biomarkers do respond rapidly upon acute changes in metabolic demand. These observations imply that quantification of redox biomarkers in a disease context also requires profiling of their temporal patterns to understand the differing trajectories upon disease-associated stressors (e.g. acute inflammatory events). Such an approach will not only inform us on the direction of the disease course in a personalized manner but also help defining patient-specific nutritional phenotypic differences and thereby allow to customize dietary advice and nutritional interventions.

Extracellular free sulfhydryl (-SH) compounds, i.e. free thiols, represent a global read-out of *in vivo* systemic redox status, not only because they rapidly scavenge reactive species but also because they act as multimodal redox relays by kinetically controlling intra- and extracellular redox exchange reactions [22,69]. Such cysteine-based *redox switches* form the central regulatory hubs of the RSI and transduce the effects of all types of reactive species into structural and functional adaptations that affect redox signaling at the whole-body level. Extracellular free thiols can be divided into proteinous and non-proteinous fractions. LMW thiols such as glutathione, homocysteine, and γ-glutamylcysteine occur in relatively low abundance in the extracellular space [69] but are nevertheless important contributors to the overall thiol redox network [70]. The steady-state concentrations of the thiol/disulfide redox couples Cys/CysSS and Trx/TrxSS, for example, have been reported to be altered in patients with IBD [[71], [72], [73]]. Homocysteine may function as integrative biomarker of the methionine recycling and one-carbon metabolism pathways (involving betaine, folate, pyridoxal-5′-phosphate, and cobalamin), while being an important nutritional precursor for RSS production. Accumulating evidence suggests that these thiol species warrant investigation in relation to disease activity, response to therapy, and prediction of disease course in IBD. In this respect, a comprehensive characterization of the thiol redox metabolome using appropriate methodological platforms [74] would be necessary to investigate their potential as integrative biomarkers in IBD.

In humans, the single reactive free thiol of serum albumin (Cys^34^) represents the main extracellular antioxidant, not only because albumin is the most abundant plasma protein but also because it has considerable transport capacity for other LMW-thiols [69,75]. Consequently, the fraction of protein-free thiols predominates (ca. 60–75% of the total circulating thiols) and is relatively more reduced (e.g. albumin, ca. 75%) compared to the pool of free cysteine which is mostly oxidized (i.e. circulates largely in the form of cystine). The dynamic interplay between reduced and oxidized forms of (proteinous) thiols can be minimally invasively, easily and robustly quantified using colorimetric or spectrophotometric assays (e.g. via 5,5′-dithiobis-2-nitrobenzoic acid, DTNB or Ellman's reagent).

In the context of IBD, the extracellular free thiol status may be viewed as an intermediate redox buffer that connects the intestinal supply of RSI precursor substances with the vast repertoire of biological thiol targets [21]. In the inflamed intestinal mucosa, continuous exposure to large amounts of generated reactive species shifts the equilibrium of reduced (free thiols) vs oxidized (disulfide) thiols towards the latter as local antioxidants get consumed. Subsequently, physiological gastrointestinal functions such as nutrient absorption, gut motility, and host-microbe interactions become disrupted as a result of oxidative damage to the tissue, cells, and individual molecules [41,43]. Thus, total free thiol status captures the balance between total oxidative burden and systemic antioxidant capacity. Previous studies have shown reduced levels of systemic free thiols in both CD and UC and inverse associations between systemic free thiols and measures of biochemical (e.g. *C*-reactive protein [CRP] or fecal calprotectin [FCal]) and endoscopic disease activity [76]. Although these observations still need validation, they demonstrate the utility of quantifying free thiols in a minimally invasive and reproducible fashion, which has the potential to become a monitoring tool for disease activity in IBD. Moreover, free thiols have shown to be receptive to therapeutic modulation by nutritional intervention in patients with IBD [77]. Modulation of the human redox system by exogenous administration of antioxidants, however, should be carefully implemented to avoid disturbances in physiological redox signaling. As such, therapeutic targeting should be reserved for patients with active IBD, as exemplified by high levels of inflammation, oxidative stress and microbial disturbances, since in the absence of inflammation or in healthy circumstances, effects of such interventions are often reported to be only marginal [78]. A promising approach for restoring redox balance in IBD is pharmacological targeting of two signaling pathways of major physiological importance: HIF-1α and NRF2/KEAP1, the critical orchestrators of hypoxia- and oxidative stress-induced cellular response, respectively.

## 4 Pharmacological modulation of HIF-1α and NRF2/KEAP1 pathways in IBD

### 4.1 Significance of the HIF-1α pathway

**Fig. 2:**
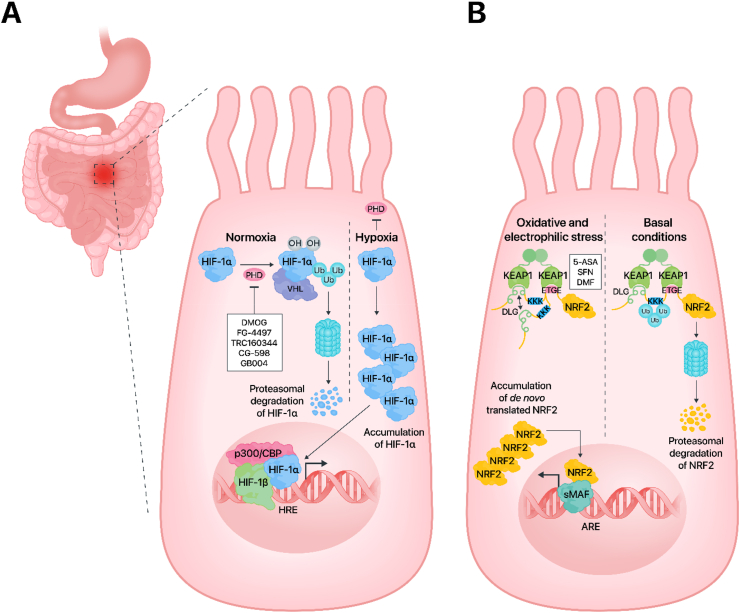
**Modulation of HIF-1 and NRF2 signaling pathways.** (**A**) Prolyl hydroxylases (PHD) are oxygen concentration-dependent enzymes, which under normoxic conditions hydroxylate the α-subunit of HIF-1 to initiate its immediate proteasomal degradation. Hypoxia and PHD inhibitors block the activity of PHDs (by reducing the hydroxylation step), which leads to HIF-1α stabilization and nuclear translocation. The complex comprising a HIF-1α/β heterodimer and p300/CBP drives the expression of hypoxia-inducible target genes. Albeit intestinal epithelial cells are shown, it is important to note that HIF is not solely restricted to these cells but is also active in immune cells like macrophages, dendritic cells, and T- and B-lymphocytes (**B**) Under non-stressed conditions, the binding of NRF2 to KEAP1 exposes NRF2 lysines for CUL3-dependent ubiquitination, which targets NRF2 to proteasomal degradation. In the presence of oxidative stress and/or electrophiles, such as 5-ASA, SFN and DMF, conformational changes prevent NRF2 ubiquitination. *De novo* synthesized NRF2 translocates into the nuclei and triggers transcription of its target genes. Abbreviations: 5-ASA, 5-aminosalicylic acid; ARE, antioxidant response element; DLG, a low affinity binding motif for KEAP1; DMF, dimethyl fumarate; DMOG, dimethyloxallyl glycine; ETGE, a high affinity binding motif for KEAP1; HIF-1, hypoxia-inducible factor 1; HRE, hypoxia response element; KEAP1, Kelch-like ECH-associated protein 1; NRF2, nuclear factor erythroid 2-related factor 2; p300/CBP, acetyltransferases p300 and CREB binding protein; PHD, prolyl hydroxylase; SFN, sulforaphane; sMAF, small musculoaponeurotic fibrosarcoma protein; Ub, ubiquitin; VHL, von Hippel-Lindau tumor suppressor protein.

By definition, hypoxia is a condition in which tissue oxygenation is insufficient to maintain adequate oxygen utilization and thus cellular bioenergetics and tissue homeostasis. In contrast to ambient air (21 kPa) and tracheal levels (19.9 kPa), the oxygen pressure in capillaries is only ∼5 kPa whereas at the level of the mitochondria it can be as low as 1 kPa [79]. Intestinal tissue oxygenation varies with local blood flow and mitochondrial activity. The oxygenation of the healthy human intestinal mucosa is, on average, between 0.1 and 5 kPa across different locations within the intestines [80]. Local oxygen concentrations differ along the crypt-villus axis, with the lowest pressure found at the villus tip [80]. However, oxygen levels in the gut lumen are far from static but undergo considerable variation upon the swallowing of liquids and food, creating steep waves with spikes of relative hyperoxygenation followed by longer periods of hypoxia/near anoxia. This ebb and flow in intraluminal oxygen concentration is known to shape the redox relationships between the human intestine and the gut microbiome [81]. On a molecular level, a central regulator of the cellular response to hypoxia is the hypoxia-inducible factor (HIF) [82]. HIF is a heterodimeric transcription factor comprising of α and β subunits (Fig. 2A). The β-subunit is constitutively expressed (and localized) in the nucleus regardless of oxygen concentration, while the amount and subcellular localization of the α subunit are tightly regulated by the oxygen levels within the cell [82,83]. Three different HIF isoforms vary in their oxygen-sensitive subunit α. HIF-3 is considered a negative regulator of the hypoxia response pathway, while HIF-1 and HIF-2 are recognized as transcriptional activators [82,84].

During normoxia, HIF-1 remains transcriptionally inactive because its α-subunit is continuously hydroxylated at the proline 402 and 564 residues by prolyl hydroxylases (PHDs) [85], which targets HIF-1α to proteasomal degradation (Fig. 2A). The hydroxylation reaction depends on the presence of oxygen, requires ferrous iron and ascorbic acid as cofactors, and is coupled to the conversion of α-ketoglutarate to succinate, releasing CO_2_ as a byproduct [86]. Three HIF prolyl hydroxylases known as PHD1, PHD2, and PHD3 can catalyze this process. Interestingly, each PHD enzyme has a different effect on the various isoforms of HIF. While PHD2 has a more powerful impact on HIF-1α than HIF-2α, PHD1 and PHD3 mostly influence HIF-2α [82,87]. The hydroxylated HIF-1α binds to the VHL (von Hippel-Lindau) tumor suppressor protein, followed by ubiquitination by the E3 ubiquitin ligase, resulting in degradation of the formed protein complex in the proteasome [88]. In addition to proline residues, asparagine 803 is also hydroxylated by the Factor inhibiting HIF-1 (FIH) [89]. Under hypoxic conditions, hydroxylation of HIF-1α is inhibited due to the inactivation of the enzymes PHD and FIH, whose activity depends on the presence of oxygen. This results in HIF-1α stabilization and accumulation, with subsequent translocation of this subunit to the nucleus. There, HIF-1α heterodimerizes with the β-subunit, followed by the addition of the coactivator acetyltransferases p300/CBP. The resultant protein complex binds to the HIF-1 consensus sequence, hypoxia response element (HRE), and initiates the transcription of target genes that are primarily involved in erythropoiesis (erythropoietin; EPO), angiogenesis (vascular endothelial growth factor; VEGF), and metabolism (glycolytic enzymes) [82] (Fig. 2A).

The GI tract is covered with a unique layer of tissue characterized by a low level of oxygen and the presence of large bacterial communities collectively referred to as the gut microbiota. The vast majority of these bacterial communities consist of obligate anaerobes, which are essential for proper intestinal metabolism and immune function [90]. Furthermore, an exclusive oxygen gradient is present in the colon, from the anaerobic lumen through the epithelium to the highly vascularized subepithelial mucosa [90]. Therefore, intestinal epithelial cells are localized within a hypoxic environment, where stabilization of HIF occurs, and which plays a central role in their physiology [90,91]. Several studies have indicated that HIF-1α activation provides a barrier-protective strategy [[91], [92], [93]]. In the GI tract, goblet cells, which are specialized epithelial cells, maintain mucin production and secretion, thus creating a protective layer. Accumulating evidence has linked HIF signaling to mucosal barrier function through transcriptional control of *MUC2*, *MUC3*, and intestinal trefoil factor (ITF) [[94], [95], [96]]. Concurrently, it was shown that mice lacking HIF-1α possess less organized and diffused mucin granules, suggesting a critical role of HIF-1α in mucin processing [97]. Of note, HIF-1 stabilization also promotes the transcription of crucial junctional proteins such as junctional adhesion molecule A or claudin-1 [98,99].

These examples of enhanced barrier function by HIF-1 emphasize its direct contribution to the preservation of intestinal epithelial integrity. Importantly, in the context of IBD, the integrity of tight junctions is altered, increasing the permeability of the epithelial barrier and leading to increased inflammation [100]. In particular, HIF-1 and HIF-2 can compete and exert opposite effects during intestinal inflammation. In IBD, HIF-1α is recognized as a protective factor since it helps preserve the functions of the intestinal barrier during colitis through the expression of barrier-protective genes, such as ITF and CD73 [96,101]. Furthermore, HIF-1α initiates an antibacterial response by raising defensins in the inflamed mucosa [102,103]. Mice with HIF-1α deletion in intestinal epithelial cells are more susceptible to intestinal inflammation and have more severe symptoms, such as increased inflammation, colon shortening, and weight loss [101]. On the contrary, HIF-2 exacerbates colitis by inducing the release of tumor necrosis factor-alpha (TNFα) in epithelial cells [104]. It is also well recognized that IBD is associated with dysregulated immune responses in the intestinal mucosa and that infiltration of immune cells modulates disease states. Mice with HIF-1 deletion in dendritic cells exhibit more severe symptoms than wild-type animals in DSS-induced colitis [105]. Moreover, deletion or constitutive activation of HIF-1 in mouse intestinal epithelial cells revealed that constitutive activation of HIF-1 is protective against trinitrobenzene sulfonic acid (TNBS)-induced colitis, while deletion of HIF-1 exacerbated the disease [106]. Similar conclusions pointing to the protective effects of the HIF pathway in IBD were drawn from *Vhl-* [101] and *Phd1*-knockout [107] mice.

Therefore, activation of HIF-1-dependent adaptive gene expression through inhibition of PHD enzymes appears to be an effective clinical strategy in the context of IBD. In mouse models of colitis, pan-PHD inhibitors such as dimethyloxalylglycine (DMOG), FG-4497, and TRC160334 were shown to be protective [[108], [109], [110]]. A phase 2 randomized multi-center clinical trial to evaluate TRC160334 in active ulcerative colitis started in 2019 in India, albeit the results have not been published yet. In a mouse model of dinitrobenzene sulfonic acid (DNBS)-induced colitis, CG-598, a new HIF-1 stabilizer, was shown to enhance the integrity of the epithelial barrier and decrease intestinal inflammation [91]. Another example of a HIF-pathway targeting compound is AKB-4924 (currently known as GB004), which proved sufficient to prevent inflammation in a TNBS model of colitis [111]. GB004 reduced the inflammatory response evoked by TNBS-induced intestinal barrier permeability by stabilizing intestinal HIF-1α [112]. The phase one clinical trial of GB004 for the treatment of ulcerative colitis demonstrated no serious adverse events and the proportion of patients achieving the endpoints was higher for GB004-treated patients compared to placebo [113]. A phase two study testing GB004 in patients with ulcerative colitis was initiated, but was announced to be terminated due to a suggested lack of treatment benefit (yet unpublished results). Importantly, PHD inhibitors that strongly block all three PHD isoforms might not be the best treatments for IBD because PHD-3 is essential for proper gut function [114]. Moreover, DMOG blocks PHD-1, which encourages NFκB-dependent inflammatory activation [115]. Therefore, the development of isoform-specific PHD inhibitors, activating HIF-1 but not HIF-2, is the next logical step in the field of IBD. PHD2 could be considered a promising target, as it acts mostly on HIF-1α [87]. Although its transcript level (*EGLN1*), similarly to PHD3 mRNA level (*EGLN3*), is much lower in intestinal cells compared to PHD1 transcript level (*EGLN2*) [116], PHD2 protein, unlike PHD1 and PHD3, is high in this organ (genecards.org). On the other hand, PHD1 KO-mice are protected from DSS-induced colitis, which is neither the case for heterozygotic PHD2 knockouts [107] nor for conditional intestinal epithelial cell-specific PHD2 knockouts [117]. This field therefore requires further detailed studies to understand this complex molecular regulation.

### 4.2 Significance of the KEAP1-NRF2 pathway

One of the first lines of defense against the overproduction of ROS is the activation of the nuclear factor erythroid 2-related factor 2 (NRF2) transcription factor, which is encoded by the *NFE2L2* gene [29,118,119]. NRF2, the ‘molecular guardian’ of cells, protects them from oxidative damage through the upregulation of a battery of antioxidative and cytoprotective genes. NRF2 belongs to the Cap'n’collar (CNC) family of proteins, which contain a characteristic structural element (the so-called basic leucine zipper, bZip) that upon interaction with other Zip partners, such as MAFs F, K, or G, enables binding to nuclear DNA at a specific enhancer, termed Antioxidant Response Element (ARE) [120]. In the molecular structure of the NRF2 protein, seven homologous functional domains (Neh1-Neh7) can be distinguished. The Neh1 domain is the DNA-binding domain, whereas Neh2 binds KEAP1 (Kelch-like ECH-associated protein 1), a repressing protein. The Neh3 domain is a coactivator, while Neh4 and Neh5 are transcription transactivation domains. The Neh6 domain is a serine-rich domain involved in the regulation of NRF2 degradation in response to phosphorylation by glycogen synthase kinase (GSK) followed by ubiquitination by the E3 ligase adapter beta-TrCP [30,121]. The primary function of the NRF2 protein in the cell is the activation of genes that encode antioxidant enzymes (the so-called phase II enzymes). Glutathione-*S*-transferase (GST), heme oxygenase-1 (HO-1), NADP(H) quinone oxidoreductase 1 (NQO1), and catalase (CAT) all belong to this group [122,123]. Under normal (unstressed) conditions, NRF2 is localized in the cytoplasm and binds KEAP1 with DLG and ETGE motifs (Fig. 2B). The ubiquitin-proteasome system degrades NRF2 at this state via a cullin3-E3 dependent mechanism. Cullin 3 (CUL3), a well-known E3 ubiquitin ligase complex subunit, binds directly to the KEAP1 protein which redirects the complex to the proteasome [124,125].

Increasing ROS levels or exposure to electrophilic activators such as sulforaphane, dimethyl fumarate, curcumin, resveratrol, triterpenoids, and quercetin, among many other molecules, modify KEAP1 cysteine residues and can thereby disable NRF2 binding. *De novo* translated NRF2 translocates to the nucleus [[126], [127], [128], [129]] where it heterodimerizes with the small MAF (sMAF) proteins, and this protein complex binds to the NRF2 consensus sequence, the ARE, located in the promoter region of target genes [130] (Fig. 2B).

NRF2 is crucial for gut formation. It influences the NOTCH and WNT signaling pathways, which drive epithelial cell differentiation during intestinal development [131,132]. Constitutive activation of NRF2 signaling in the intestinal epithelium results in extended intestines and increased enterogenesis. This is achieved by suppressing the NOTCH downstream effector MATH1 in enterocyte progenitor cells [132]. On the other hand, Nrf2 deficiency results in the formation of a longer colon, a different distribution of crypts, and the enlargement of goblet cells with a noticeably higher level of mucin 2 [131]. Thus, the NRF2/KEAP1 axis regulates GI tract development and function and may, therefore, also modulate the progression of IBD. Compared to wild-type mice, NRF2 knockout mice were more susceptible to DSS-induced colitis, characterized by enhanced rectal bleeding, colon shortening, crypt overgrowth, and increased immune cell infiltration [133]. Furthermore, mice lacking NRF2 display severe oxidative damage upon treatment with DSS [133]. However, experiments using genetically modified mice expressing constitutively active NRF2 selectively in epithelial or myeloid cells revealed an aggravated DSS-induced acute colitis phenotype. In contrast, in the IL-10 KO-model of spontaneous chronic colitis, this effect was absent [134].

The hormetic characteristics of NRF2 activity, i.e. demonstration of a biphasic (or triphasic) biological response to exposure to increasing NRF2 activity, have been shown in several animal experimental models [135,136]. Experimental data indicate a dual role of NRF2 in IBD development, since both NRF2 deficiency and NRF2 overexpression can aggravate the disease phenotype. Nevertheless, a moderate degree of activation of this pathway might be a reasonable approach for therapy design. One example is mesalamine, also known as 5-aminosalicylic acid (5-ASA), which is commonly used as a medical treatment for patients with IBD, particularly those with UC [137]. The oxidized form of 5-ASA is electrophilic. It activates the NRF2-HO-1 pathway mediating the anti-inflammatory effects of this drug in the gut [138]. Interestingly, NRF2 activation by sulforaphane (SFN), a naturally occurring electrophilic isothiocyanate, was shown to protect against experimentally-induced colitis in rats via different mechanisms, such as enhancing antioxidant activity, mitochondrial biogenesis, and inhibiting DNA polymerization [139]. More recently, SFN was found to ameliorate DSS-induced colitis through modulation of gut microbiota composition and increased expression of tight junction proteins such as ZO-1, occludin, and claudin-1, with concomitant reduction of pro-inflammatory cytokines [140]. Moreover, a natural herb called *Perilla frutescens* has anti-inflammatory and antioxidant properties as its administration during DSS-induced colitis in mice inhibited NF-kB and STAT3 pathways and increased NRF2 and HO-1 levels [141]. Carnosic acid, another plant-derived molecule, demonstrated antioxidant and protective effects in DSS-induced murine colitis by inhibiting DSS-induced inflammasome activation and regulating the NRF2-mediated oxidative stress response [142]. Another example is electrophilic dimethyl fumarate (DMF), which has anti-inflammatory and antioxidant properties in various inflammatory diseases. DMF induced the activation of the NRF2-ARE pathway in the DSS-induced colitis mouse model, resulting in the upregulation of its target antioxidant enzymes HO-1 and NQO1 [143]. DMF protected human intestinal epithelial cells from H_2_O_2_-induced barrier dysfunction by restoring tight-junction proteins ZO-1 and occludin expression via the NRF2-HO-1 pathway [144]. Finally, DMF reduced DSS-induced colon inflammatory damage by increasing glutamate-cysteine ligase catalytic subunit (GCLC) protein levels, decreasing COX-2, and increasing glutathione peroxidase (GPX) expression [143]. According to these findings, NRF2 activators may limit the progression of IBD in the early stages by inhibiting inflammation and oxidative stress. However, it should be noted that NRF2 activators may also exert nonspecific effects related to their off-targets [128].

## 5 Clinical implementation of redox medicine in IBD: challenges and perspectives

Currently, somewhat outdated theory/symptom-based classifications and predictions for the management of IBD govern daily clinical practice, largely ignoring the complex, heterogeneous and multifactorial nature of the disease. For example, the Montreal disease classification, used to define subgroups of patients based on location, behavior and extent of the disease, fails to reliably predict disease course and prognosis and has poor utility in determining the optimal timing and most appropriate treatment strategy for patients [145]. This is where an unbiased, data-driven, machine learning-based approach could help by interrogating the vast amount of molecular data in conjunction with patient-specific disease characteristics, provided that it is used against an overarching conceptual framework such as that offered by the RSI. With the advent of multi-omics (e.g. genomics, transcriptomics, and proteomics) profiling technologies, a *systems biology* approach is increasingly advocated to unravel disease pathogenesis and suggest alternative approaches to disease management [146]. Systems biology refers to the holistic and mathematical modeling of complex biological systems [24]. In recent years, systems biology approaches have advanced rapidly mainly due to unprecedented computational and technological developments [25], allowing the construction of *integrative multi-omics* models [147,148]. These efforts have resulted in ‘big data’ and enabled the construction of large molecular datasets for biomarker discovery. While such molecular constructs have also been established for IBD, data are sparse and challenges remain with regard to independent validation and integration with clinical phenotypes/outcomes. Most of the variation in molecular data is explained by inter-individual differences, which can mask potentially relevant observations [149].

**Fig. 3:**
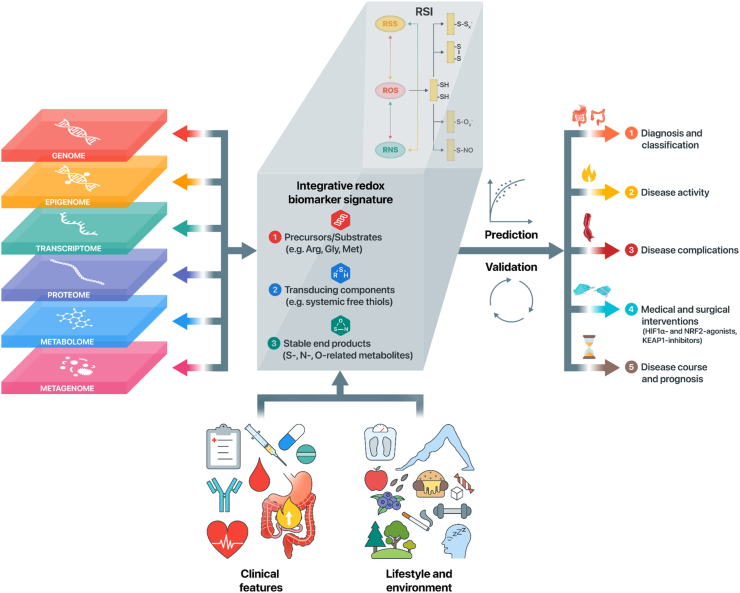
**Personalized redox medicine and redox metabolomics in the context of IBD.** An unbiased, data-driven multi-omics approach may help to define the key components of an integrative redox biomarker signature, while taking into account clinical features and lifestyle-related and environmental factors. Integrative redox-omics, i.e. analyzing redox biomarkers across different levels of biological organization, may help identify the key biomarkers based on the RSI. While integrating all this information, however, it is critical to allow careful phenotypic patient stratification at the same time. After the identification of predictive redox biomarker signatures, validation (both internally and externally) in independent cohorts should be pursued to reliably evaluate their utilities. Ultimately, this may lead to outcome-specific biomarker signatures that may help to (1) aid in diagnosis and improve molecular reclassification of IBD (e.g. disease location or extent), (2) to discriminate between quiescent and active disease states, (3) to discriminate between different types of disease complications (e.g. stricturing and penetrating disease phenotypes), (4) to predict individual responses to medical and surgical interventions in IBD, and (5) to help predict the risk of post-surgical disease course and, eventually, disease prognosis. Abbreviations: Arg, arginine; Gly, glycine; Met, methionine; RSI, Reactive Species Interactome.

A tangible -omics approach to redox biology or *redox metabolomics* does not yet exist, although efforts are underway to develop this and get a better grasp of the dynamics and architecture of the human redox system. Redox metabolomics can be defined as the analysis of stable products of redox-driven metabolic pathways [150]. Until now, however, the development of methodological approaches in this field is lagging behind those in other areas. Presumably, this is so because a distinct set of criteria that redox biomarkers need to fulfill to comprehensively assess the redox system is missing, which is caused by the absence of accurate definitions of outcome measures that are of biological relevance in that they represent a substantial proportion of the human redox architecture [61,151]. Furthermore, methodological constraints and the requirement for highly sophisticated techniques inherent to the measurements of reactive species further contribute to the comparatively slow pace with which redox metabolomic approaches are developing [152]. Based on the principal elements of the RSI, however, the design of such an approach has recently been put forward [21,22,68] that may also hold promise for IBD (Fig. 3). This approach focuses on the core constituents of the RSI that form central regulatory ‘hubs’ of redox metabolism and are referred to as ‘integrative biomarkers’ since they may be representative of multiple redox-regulated metabolic pathways. Suggested targets for this approach capture the metabolic status of the RSI and constitute integrative measurements of (1) nutritional precursors or substrates of the RSI (e.g. H_2_S, methionine, arginine, cysteine, and cofactors), (2) cysteine-based redox switches as transducing components or the dynamic interplay between circulating oxidized and reduced as well as albumin-bound thiols) and (3) the stable end products of the RSI in the form of S-, N-, and O-derived metabolites. Examples of these metabolites have been reviewed elsewhere [150]. This aggregate of integrative biomarkers could faithfully assemble into a structured multi-omics approach to become a defining feature of redox biology and allow the reclassification of disease processes, including IBD.

As soon as well-characterized and functional redox metabolomics approaches have been established, integration of such profiling methods should progress towards implementation in clinical practice. This, however, would be a major leap forward, since the complexity and impracticality of such initial high-throughput and high-resolution methods would face several financial and logistic challenges. Instead, in the first step, one should strive for the development of easy-to-use, robust and cost-effective diagnostic and prognostic tools that could enrich the arsenal of routine biochemical/metabolic biomarkers with select redox metabolomic signatures. For instance, nanotechnological tools such as lab-on-a-chip devices could be used for the self-monitoring of disease activity (“telemonitoring”) through parallelized measurements of integrative redox biomarkers, offering a tool to identifying patients who might benefit most from redox-modulating therapeutics. Such platforms could also be created for integrative blood-based omics measurements that are incorporated into a data-driven personalized biomarker signature. Although concrete examples hereof do not yet exist, due to the described limitations, the first results from other layers of multi-omics data in IBD have shown compelling successes. The most striking example in this context is the development of a transcriptomics- and blood-based 17-gene prognostic biomarker that could reliably predict prognosis in newly diagnosed patients with IBD [153]. This CD8^+^ T-cell expression signature was able to accurately discriminate between patients who later experienced an aggressive disease course with higher frequency and severity of exacerbations and disease relapses from patients who did not and instead followed a rather mild disease course [154]. Because this biomarker signature remained robust throughout multiple prospective, independent validation studies, it led to the establishment of the first-ever biomarker-stratified clinical trial in IBD [155]. This trial is aimed at evaluating this transcriptional biomarker for its capacity to improve clinical outcomes by facilitating personalized medicine for patients with IBD. One could also think of such an approach arising from extensive redox metabolomic profiling, which may eventually help to stratify patients before the onset of redox-targeted therapy and adjust its timing, frequency and duration in a fashion tailored to each patient. A shift towards ‘clinical -omics integration’ could help guide the characterization of homogeneous subgroups of patients that might benefit from a particular treatment by comprehensive molecular and clinical profiling.

## 6 Concluding remarks and future perspectives

Disrupted redox signaling processes and ‘oxidative stress’ play a critical role in the pathophysiology of IBD, which is accompanied by intestinal inflammation and manifested by oxidative damage of intestinal tissue. Achieving a comprehensive understanding of the human redox system is essential to develop effective redox-modulating therapeutic strategies in patients with IBD. The RSI framework may help to prioritize integrative biomarkers of whole-body redox status, encompassing precursor substances, the transducing elements (cysteine-based redox switches), and stable downstream S-, N-, and O-derived metabolites. However, a critical appraisal of the utility of currently established redox biomarkers is warranted, and an unbiased, data-driven approach to finding adequate redox biomarkers (while characterizing their longitudinal dynamics and response to physiological and pathophysiological stressors) will eventually allow determination of the disease process under study and, possibly, its directionality (e.g. the future occurrence of disease flares or complications). Integrative biomarkers based on the RSI concept hold the potential to serve as a monitoring tool by interrogating an individual's redox phenotype in order to minimize interference with physiological redox signaling. Using this framework to determine the individual-specific level of “redox stresses”, therapeutic decisions could become more personalized to increase the chance of achieving whole-body redox balance and apply redox-targeted therapy. Together, these approaches may provide us with more granular insight into the multi-layered regulatory network that underpins human redox status and may foster the transition from symptom-based towards mechanism-based molecular reclassification of IBD. Two emerging therapeutic targets for IBD — HIF-1α and NRF2/KEAP1 — represent viable therapeutic options to restore aberrant redox balance and may help identify new compounds with the potential to break the therapeutic ceiling in IBD. Selective activators of HIF-1α and moderate specific activators of the NRF2 response represent major opportunities for molecular medicine in IBD therapy. This needs to be accompanied by a more nuanced understanding of disrupted redox signaling in IBD, alongside translational efforts to achieve ‘clinical -omics integration’ for redox medicine.

## Author contribution

ARB and HvG conceptualized the review. ARB, AGP and DK performed extensive literature review and wrote the first draft of the manuscript. All authors contributed to critically reviewing the manuscript, revising it for important intellectual content, and read and approved the final version of the manuscript to be submitted for publication.

## Funding

No specific funding was received for this manuscript.

## Declaration of competing interest

The authors declare that they have no known competing financial interests or personal relationships that could have appeared to influence the work reported in this paper.

## Data Availability

No data was used for the research described in the article.
